# Encoding, training and retrieval in ferroelectric tunnel junctions

**DOI:** 10.1038/srep27022

**Published:** 2016-05-31

**Authors:** Hanni Xu, Yidong Xia, Bo Xu, Jiang Yin, Guoliang Yuan, Zhiguo Liu

**Affiliations:** 1 National Laboratory of Solid State Microstructures, Collaborative Innovation Center of Advanced Microstructures and Department of Materials Science and Engineering, College of Engineering and Applied Science, Nanjing University, Nanjing 210093, China; 2 School of Materials Science and Engineering, Nanjing University of Science and Technology, Nanjing 210094, China

## Abstract

Ferroelectric tunnel junctions (FTJs) are quantum nanostructures that have great potential in the hardware basis for future neuromorphic applications. Among recently proposed possibilities, the artificial cognition has high hopes, where encoding, training, memory solidification and retrieval constitute a whole chain that is inseparable. However, it is yet envisioned but experimentally unconfirmed. The poor retention or short-term store of tunneling electroresistance, in particular the intermediate states, is still a key challenge in FTJs. Here we report the encoding, training and retrieval in BaTiO_3_ FTJs, emulating the key features of information processing in terms of cognitive neuroscience. This is implemented and exemplified through processing characters. Using training inputs that are validated by the evolution of both barrier profile and domain configuration, accurate recalling of encoded characters in the retrieval stage is demonstrated.

Ferroelectric tunnel junction (FTJ), a device that two metal electrodes sandwich a few-unit-cell ferroelectric barrier, possesses high speed switching of tunnel transmission with potentially low operation energy^1^,^2^. Tunneling electroresistance (TER), generally used to assess a FTJ, refers to the electrical switching between two resistance states (ON and OFF)^3^,^4^. The polarization reversal in ferroelectric layer gives rise to the change in distorted potential profile, on which the tunneling transmittance depends exponentially^5^,^6^,^7^. That is why the ferroelectric modulation on the barrier generates the TER in FTJs^2^,^8^,^9^,^10^,^11^,^12^. On the other hand, FTJs also promise an important degree of freedom to engineer the junction resistance in terms of the ferroelectric domain structure^13^. The voltage-controlled domain configurations support the quasi-continuous resistance variations and thereby plasticity in FTJs^13^. Such multinary feature allows ferroelectric junctions to implement not only logic operations but also neuromorphic functionalities such as brain-inspired processing and memory^12^,^13^,^14^,^15^. Among recently proposed possibilities, hardware-implemented artificial cognition has been envisioned with high hopes.

From an information processing perspective in cognitive neuroscience, encoding is the first stage in the formation of memory^16^. It refers to the processing and combining of sensory information like characters, images and sounds for complex tasks. The encoded information is then recorded in the storage stage, in which trainin g operations are always required for memory solidification, and can be recalled in response to some cue for use in some process or activity in the retrieval stage^16^,^17^,^18^. Each of these stages is indispensable to constitute the whole chain for processing and memory aiming at neuromorphic applications. Encoding devices also supplying memory could be one of the approaches. However, the present encoding devices based on logic elements only carry out the coding operations but are incapable of storage and recalling^19^.

Another way to fulfill the task is endowing store elements with encoding. This is expected to be implemented in quantum nanostructures of various tunnel junctions like FTJ owing to its multilevel memory by nature or magnetic tunnel junction, another element possessing information storage and reprogrammable logic (refs 20, 21, 22), for example, the extraordinary Hall balance element (ref. 23). In this work, we take processing some characters as an example and showcase encoding, training, memory solidification and retrieval in BaTiO_3_ (BTO) FTJs. The accuracy of processing the codes in FTJs relies on the reliable control of TER at the nanoscale. However, challenges, such as the retention properties of FTJs, remain yet to be overcome^24^,^25^. The reported poor retention and short-term store in the intermediate states in particular (ref. 26) are fatal to the cognitive operations. The decay of tunneling resistance over time will bring about failed recalling in the retrieval stage. Here we also show the training-determined memory solidification, confirmed by both piezoresponse force microscopy (PFM) and electrical transport measurements, to guarantee the accurate retrieval. This improvement of the retention for the intermediate states is one of the neural functions making up the whole chain of cognition that originates from the most basic FTJ structure and does not rely on introducing some new material or structure.

## Results

### Polarization reversal-induced resistance switching

**Figure 1:**
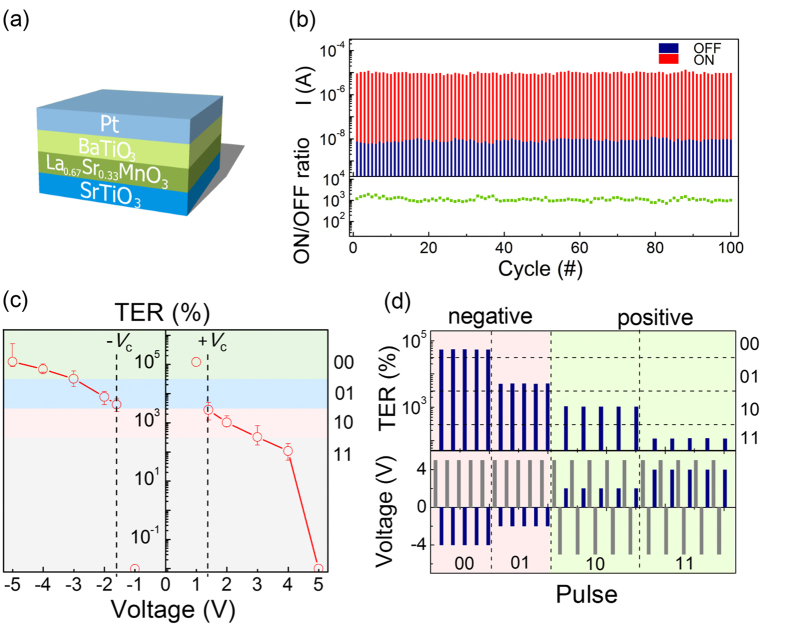
The definition of multi-number in BTO FTJs. (**a**) Schematic of BTO FTJs used to implement encoding and retrieval. (**b**) The reproducibility of FTJ devices having ON/OFF ratios around 1000, read at 0.1 V. The conductance states are switched by ±5 V writing voltages of 200 *μs* in width. (**c**) TER as a function of input voltage. The data are from the statistical results of over 50 operations for each voltage. The dash lines label the coercive voltages, +1.4 and −1.6 V. (**d**) The definition of 4 codes according to the TER values by applying −4, −2, +2 and +4 V pulses of 200 *μs* in width, respectively. The pulses in grey indicate the reference and the erase pulses of ±5 V. Five operations show the reproducibility of such definition.

The FTJs used to carry out encoding and retrieval have a sandwiched structure of Pt/BTO (2 nm)/La_0.67_Sr_0.33_MnO_3_ (LSMO)/SrTiO_3_ (STO) (Fig. 1a). This heterostructure is able to tune the tunneling transmittance by ferroelectric polarization reversal. The local ferroelectric feature of thin BTO on LSMO/STO (the high resolution transmission electron microscopy images, Supplementary Figure S1) is confirmed by PFM analysis (Supplementary Figure S2). The conductance switching by loading pulses of ±5 V is shown in Fig. 1b, where the different conductance states resulted from +5 and −5 V are denoted as ON and OFF states, respectively. The readout ON/OFF ratio, at 0.1 V, reaches as high as 10^3^ at room temperature. 100 consecutive switching cycles are summarized in Fig. 1b, demonstrating the reproducibility of our FTJ devices having ON/OFF ratios around 1000. This reproducible high ratio promises more in-between states with distinguishable differences between each other.

### The definition of multi-number in BTO FTJs

The writing amplitude regulates the conductance ratio, bearing multilevel TER in BTO FTJs. The TER is calculated from TER(*v*) = [*R*(*v*) − *R*_ON_]/*R*_ON_, where *R*_ON_ is the resistance at the ON state after applying +5 V, and *R*(*v*) is the readout resistance in response to the input voltage pulse of amplitude *v*. Every TER measurement begins with a pulse of +5 V for reference. For the TER from negative voltages, negative input is loaded directly following the +5 V pulse. However, for the TER from positive voltages, there is always an additional −5 V pulse to erase the ON state before the positive input, like the voltage pulses described in Fig. 1d. All the resistance values are read at 0.1 V after each input. Figure 1c shows the writing voltage-dependent TER. The voltages between the coercive voltages of +1.4 and −1.6 V cannot reverse the polarization, so the TER is as high as 10^5^% for positive voltages and as low as 0 for negative ones. Each input stronger than the coercive voltages, switching the polarization, maps to an output TER that ranges in degree between 0 and 10^5^. For positive inputs, the higher amplitude, the smaller TER. On the contrary, in the case of negative inputs, the higher amplitude, the larger TER. All these make it possible to define the codes in the multi-number system, according to the TER of different orders of magnitude. However, the multilevel storage is necessary but not sufficient to the encoding. The definition of codes is not an arbitrary choice of different states. There are criteria for such multi-bit system.

Four TER bands are ruled in Fig. 1c to discriminate one code from another, taking into account both the device-to-device and the cycle-to-cycle variations of TER. The inputs are grouped into 4 voltage intervals, (−5, −3], (−3, −1.6], [+1.4, +3) and [+3, +5). ±2 and ±4 V, as the kernel of each interval respectively, produce TER with differences of one order of magnitude. Given these 4 inputs, the outputs are accordingly labeled as four codes based on the TER values in descending order. All the TER values within one band, having less than tenfold difference, belong to the same code. Each defined code standing for a two-digit piece, i.e. “00”, “01”, “10” and “11”, must carry two parts of the input information so that the inputs can be accurately encoded and decoded. In the case of TER, these two parts of the input information are the polarity and the amplitude of voltage pulse. The first part indicates the polarity, “0” for the negative input, while “1” for the positive one. The second part conveys the amplitude-controlled conductance information with the same polarity (i.e. with the same first part), “1” for the input producing lower TER and “0” for the one having higher TER. For example, a −4 V pulse defines “00” and a + 4 V pulse makes “11”. Figure 1d gives the detailed definition of these 4 codes, from which they are able to express eight-digit codes such as ASCII codes.

### Encoding and retrieval in BTO FTJs

**Figure 2:**
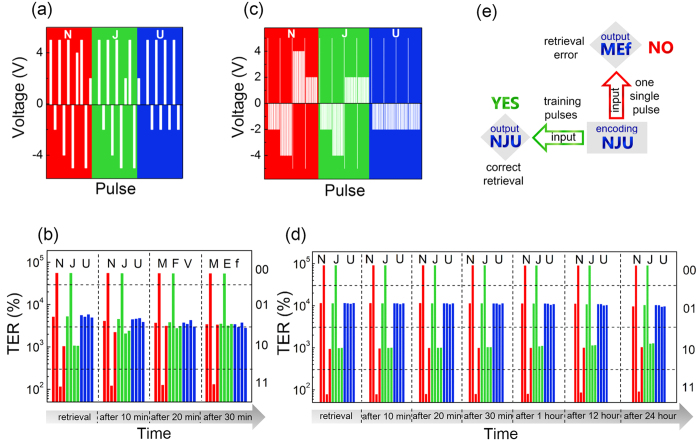
Encoding, training and retrieval in BTO FTJs. (**a**) Encoding inputs composed of single pulse with 200 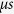 in width. The standard eight-bit ASCII codes for characters “NJU” are “01001110”, “01001010” and “01010101”, respectively. (**b**) Wrong retrieval of “NJU” over time using encoding inputs described in (**a**). (**c**) Encoding inputs composed of training pulse sets of 20 intermittent pulses. Each pulse is 200 *μs* in width and 1 ms in interval. (**d**) Accurate retrieval of “NJU” using encoding inputs described in (**c**). (**e**) Schematic diagram showing different retrieval results by two types of encoding inputs: single pulse and training pulse set.

Here the codes for characters “NJU”, the short for “Nanjing University”, are taken as an example to showcase the encoding using BTO FTJs. “N” is expressed as “01001110” in the standard eight-bit ASCII codes. Figure 2a exhibits the assembled encoding pulses for “N”. The other two characters, “J” and “U”, are encoded in the same way, as also shown in Fig. 2a.

The leftmost column of Fig. 2b gives the retrieval results of the coded “NJU” just after the encoding stage. The TER values, read at 0.1 V, confirm the readout of “NJU” and thus correct retrieval. Then such retrieval operation is done once again after a waiting time of 10 min. The readout codes remain 01001110(“N”), 01001010(“J”) and 01010101(“U”). However, TER changes as time elapses, particularly the values for “01” and “10”, although they keep inside their original bands. The drastic changes in TER for “01” and “10” appear after 20 min and longer. Some of TER values for “01” drop into band “10”, while some for “10” cross the boundary into band “01”. The retrieval of correct characters fails because the “NJU” turns into “MFV” after 20 min and “MEf” after 30 min. The failure in retrieval comes from the wrong readout of “01” and “10”, since “00” and “11” barely changes over time in Fig. 2b. The fact that “01” and “10” decay more than “00” and “11” does indicate the poorer retention of TER and short-term store from lower writing amplitude of ±2 V.

### The variations in barrier profile over time

**Figure 3:**
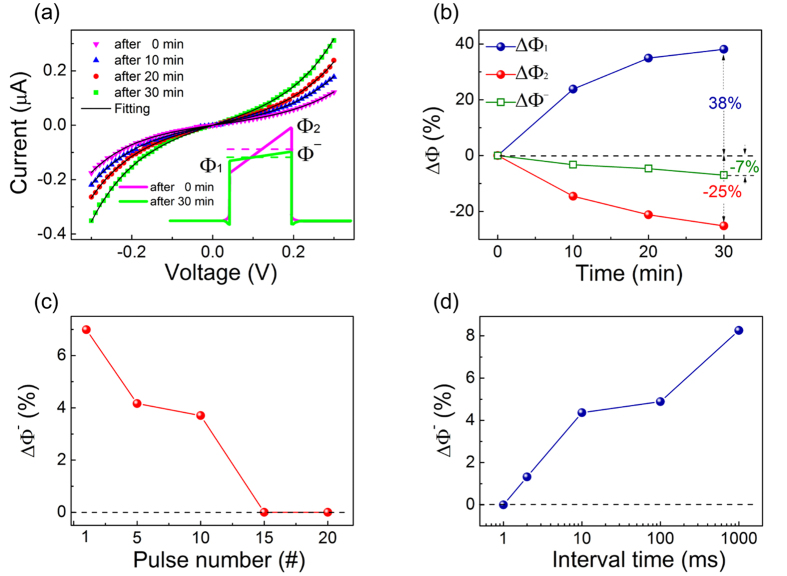
Different variations in barrier profile over time after encoding “01” by two types of inputs. (**a**) The evolution of *I*-*V* curves after encoding by a single pulse of −2 V in amplitude and 200 *μs* in width. Solid black lines showing the fitting of the experimental data according to the WKB model. The inset schematically depicts the change in potential energy profile over time. (**b**) The change in potential energy over time. All the ΔΦ_1_, ΔΦ_2_ and ΔΦ^−^ data are expressed as ΔΦ(*t*) = [Φ(*t*) − Φ(0)]/Φ(0), where Φ(0) is the initial potential energy right after the encoding and Φ(*t*) is the one at time *t*. (**c**,**d**) The suppressed ΔΦ^−^ after encoding “01” by input composed of a training pulse set. The amplitude and the duration for all pulses in the set are −2 V and 200 *μs*, respectively. The pulse number ranges from 1 to 20 while the time interval remains 1 ms in (**c**). The time interval decreases from 1000 to 1 ms while the pulse number is 20 in (**d**). ΔΦ^−^ in (**c,d**) is the change of Φ^−^ after 30 min, i.e. ΔΦ^−^(30 min) = [Φ^−^(30 min) − Φ^−^(0)]/Φ^−^(0).

The decay of “01” is exemplified herein to illustrate the origin of the wrong retrieval in Fig. 2b. The tunneling current and the TER are functions of the distorted potential barrier at interfaces. The change in barrier profile over time determines how TER responds to time. The barrier Φ_1_ (between BTO and LSMO) and Φ_2_ (between BTO and Pt), and the average potential height Φ^−^ = (Φ_1_ + Φ_2_)/2 as a result, can be extracted from the experimental tunneling current-voltage characteristics (*I*-*V*) in the light of WKB approximation^27^ (Supporting Information). The evolution of *I*-*V* after encoding “01” by pulse of −2 V is shown in Fig. 3a. All the curves follow the WKB model, from which the corresponding barrier parameters are extracted. Figure 3b summarizes the changes in these parameters over time. Φ_1_ increases with time but Φ_2_ decreases in the meantime. After 30 min, the residual Φ_2_ is only 75% of its initial value and Φ_1_ is increased by 38%, leading to the less distorted potential profile. The average potential height Φ^−^ lowered by about 7% explains the drop of tunneling resistance.

The transformation in trapezoidal potential barrier stems from the relaxation of polarization or the dissipation of oxygen vacancies accumulated at interface. Oxygen vacancies compensating the negative bound charges of polarization are believed to reduce the average barrier height and decrease the junction resistance^25^. Removing them from interface should recover the barrier and thereby increase the resistance^25^. But it is not the situation here. After loading a −2 V input, Φ^−^ decreases (Fig. 3b) and accordingly the TER drops (Fig. 2b and Supplementary Figure S3) as time elapses. It is consequently not an oxygen vacancies-dominated behavior, leaving the relaxation of upward polarization responsible for the decay of “01”. The time-dependent PFM images (Supplementary Figure S4) confirm the metastable domain configurations switched using −2 V.

### Domain relaxation behaviors

Ferroelectric switching occurs following the kinetics of nucleation and growth. The accumulation of stress or depolarization field brings about the thermodynamic instability destabilizing the domain in FTJs^28^. The strength of depolarization field is basically a function of polarization value in the light of the electrostatic calculations on the capacitor geometry^29^. Higher voltage input yielding larger polarization could give rise to a stronger depolarization field than lower input does. As a result, rather than in “01” and “10”, intense decay is expected to take place in “00” and “11”. However, it is contrary to our case, as demonstrated in Fig. 2b and the statistical data shown in Supplementary Figure S5. The effect of depolarization field cannot be completely ruled out because it is intrinsic in FTJs^29^,^30^,^31^, but it does not play a leading role in the decay behaviors here.

The cumulative stress from the nucleation and growth of up domains inside the initial down domains creates the metastable situation. Given that the polarization is not fully switched during the encoding by −2 V pulse (Supplementary Figures S4 and S6), the stress arisen between the switched and the non-switched domains results in the thermodynamic instability. After removing the voltage, relaxation takes place till a new thermodynamic equilibrium between them is reached^28^. On the one hand, it reduces the size of up domains and even reverses some of the switched domains back to the initial orientation, where the degraded TER is thus inevitable. On the other hand, the residual nuclei in the new equilibrium support the subsequent formation of up domains once successive pulses of −2 V are imposed at appropriate intervals. Although relaxation also occurs, it should be suppressed step by step with the increasing repetition. In addition, the increasing repetition also provides multiple opportunities to select the switching path more than one single stimulus, considering the coexistence of several switching paths with different stress or defect situation^32^.

**Figure 4:**
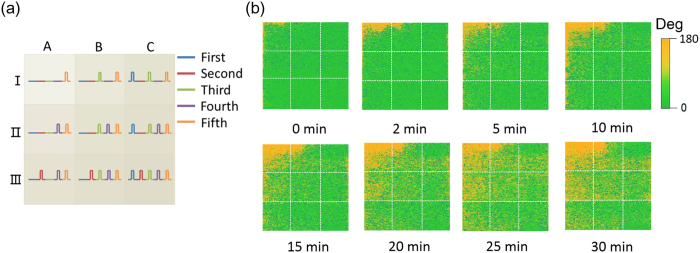
Different domain relaxation behaviors depending on the number and interval of the applied inputs. (**a**) Schematic of a designed 3 × 3 pattern for PFM experiments. The illustrated pulses in different colors indicate the loading sequence of writing by −2 V. The cumulative number and the time interval of writing inputs change from one square to another. (**b**) PFM mappings monitored at different time ranging from 0 to 30 min after completing the pattern in (**a**). Up and down domains are in green and yellow respectively. Different responses to time are observed in these nine regions.

The stimuli repetition-dependent relaxation behaviors are shown in Fig. 4b to give a direct perception. The PFM images in a designed 3 × 3 pattern, having 9 squares with the total area of 1.5 × 1.5 *μm*^2^, are monitored at a randomly chosen location on BTO film. Each square, occupying one ninth of the total area, is electrically written with different history from one another, as schematically described in Fig. 4a. Both the number of stimuli and the time interval between them change from one square to another. For example, regions IA, IB and IC have different stimulus number, while regions IB and IIA have the same number of 2 stimuli but different intervals. To achieve such 3×3 pattern, five steps are implemented in sequence (Supplementary Figure S7). First, column C is written by the tip applying −2 V, but the other two columns A and B are programmed with 0 V in the meantime. The stimulus number for regions IC, IIC and IIIC is 1 but 0 for other regions. Second, the row III is programmed by −2 V but 0 V is applied to the rest, I and II. The overlap region IIIC is then written twice whereas the stimuli in other regions are only one time or none. Third, the regions to which −2 V signal is applied are columns B and C. Fourth, the applied voltage for rows I, II and III is 0, −2 and −2 V, respectively. The final step is to program the whole 9 regions using −2 V. As a result, the number of stimulus changes from 1 (IA) to 5 (IIIC). Different time intervals are also accomplished in those regions with the same number, such as the diagonal “IC → IIIA” showing the variation of time intervals.

Right after programming the pattern of Fig. 4a, PFM mappings are recorded at different time nodes to demonstrate the evolution of domain configurations in these 9 regions. Different relaxation behaviors are found from one square to another in Fig. 4b as expected. There is drastic and fast relaxation within 30 min in region IA that is programmed one time only. But things are different in the regions written more than once, especially region IIIC in which the relaxation is greatly suppressed. Experimental and theoretical studies have given the evidence of surface ionic adsorption contributing to the stable polarization direction^31^. For instance, OH adsorbates are adequate to stabilize the upward polarization. However, the improved stability observed in region IIIC is not a result of ionic adsorption because these nine regions are exposed to the identical environment during the whole PFM experiments. The only difference between them is the number and interval of the applied inputs. The detailed PFM images with higher resolution showing different relaxation behaviors are listed in Supplementary Figure S6. We also performed the domain evolution on samples with Pt top electrodes. The same stimuli repetition-improved relaxation behaviors are observed (Supplementary Figures S4 and S6), indicating such phenomenon does not depend on whether the samples with or without top electrode.

## Discussion

The PFM results shown in Fig. 4 (and Supplementary Figures S1 and S6) point out a reasonable way to improve the retention depending on not only the number but the interval of the encoding inputs, resembling the memory solidification that is more sensitive to the frequency of the stimulus rather than the duration^33^,^34^. The recovered barrier profiles by regulated input number and interval corroborate its validity. Figure 3c,d give the ΔΦ^−^ as functions of pulse number and interval, respectively. All the ΔΦ^−^ values are recorded 30 min after the encoding. Φ^−^ hardly changes when the encoding is trained by a pulse set of 20 in number and 1 ms in interval. To further confirm the superiority of such pulse set of intermittent input pulses over one continuous pulse (even with longer duration), encoding by a single pulse of 4 ms (equivalent to the total integrated excitation time of 20 pulses of 200 *μs*) is also carried out. Such single long pulse is incompetent in encoding and further recalling because it leads to the lower Φ^−^ after 30 min (Supplementary Figure S8).

Following this principle, the characters “NJU” are encoded using a training input set composed of 20 pulses with 200 *μs* in duration and 1 ms in interval (Fig. 2c). The readout codes barely change with time (Fig. 2d). They maintain 01001110(“N”), 01001010(“J”) and 01010101(“U”) after 24 hours. The statistical data of this accurate retrieval are provided in Supplementary Figure S5 to show the repeatability and reliability of this approach. As a result, we here present clear experimental evidence of encoding and retrieval functionalities in BTO FTJs, which enable FTJs to supply the key features of processing and memory in cognitive neuroscience. The enhanced stability of TER by fine control of training inputs, especially the TER from those in-between states, offers a promising solution to the long sought improved retention in FTJ applications. Such memory solidification by improving the stability of the retention in FTJs is one of the basic neural functions where there is no special requirement of any new structure into the systems. Future experimental and theoretical studies on the detailed interface structure and domain evolution may help further elucidate this behavior. Given the reproducible multilevel TER with improved retention and reliability, FTJs are allowed to support other complex tasks. For example, in the light of both our previous memristive “abacus” approach (ref. 35) or the three dimensional magnetic abacus concept (ref. 36), the brain-inspired computation will be accomplished.

## Methods

The BTO (2 nm)/LSMO (10 nm) heterostructures were grown on single-crystal (001) STO substrates by pulsed laser deposition using a KrF excimer laser (COMPex, Lambda Physik, 248 nm in wavelength and 30 ns in pulse width). The laser energy density and repetition rate were 2.5 Jcm^−2^ and 1 Hz, respectively. LSMO films were deposited at 780 °C with an oxygen pressure of 15 Pa. After that BTO films were deposited at 780 °C with an oxygen pressure of 10 Pa. The samples were then annealed in oxygen atmosphere (10^4^ Pa) at 750 °C for 20 min.

The cross-sectional morphology of the heterostructures (Supplementary Figure S1) was investigated by high resolution transmission electron microscopy (Tecnai G^2^F20 TEM) operated at 200 kV. The local ferroelectric properties and current mapping were measured using an Asylum Research Cypher scanning probe microscope. Olympus AC240TM Pt/Ti-coated silicon cantilevers were adopted in the PFM measurements. Hysteresis loops were collected in the DART (dual a.c. resonance tracking) mode with a triangular pulse applied to the tip. Phase and amplitude images were recorded in single-frequency PFM mode. Current mapping was recorded using conductive-diamond-coated silicon cantilevers (CDT-NCHR, NanoWorld).

Pt top electrodes with 50 nm in thickness and 100 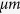 in diameter were sputtered through a shadow mask at room temperature. All the electrical measurements were carried out in a closed Cascade Summit 11000 M probe station that is equipped with Keithley 4200-SCS semiconductor characterization system (for encoding and retrieval experiments and *I*-*V* characterization) and Keithley 2400 source-measure unit (for cycle measurements). All the pulses were applied to the Pt electrodes while the LSMO electrodes were always grounded. The pulses used in encoding were 200 *μs* in width. After the encoding by inputs composed of single pulse or training pulse set, TER was recalled using a readout pulse of 0.1 V in amplitude and 200 *μs* in width.

All the measured I-V curves follow the WKB model assuming a tunneling current through a trapezoidal potential barrier depending on the polarization pointing. The current density is expressed as


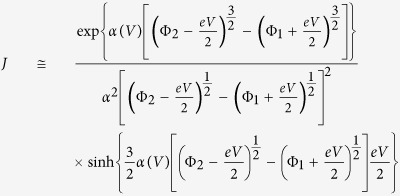


where 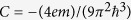, 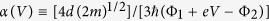, Φ_1_ is the barrier height at BTO and LSMO interface, Φ_2_ is the barrier height at BTO and Pt interface, and *d* is the film thickness^9^.

## Additional Information

**How to cite this article**: Xu, H. *et al.* Encoding, training and retrieval in ferroelectric tunnel junctions. *Sci. Rep.* **6**, 27022; doi: 10.1038/srep27022 (2016).

## Supplementary Material

Supplementary Information
