# Supplementary material for: Encoding, training and retrieval in ferroelectric tunnel junctions
Source: Sci Rep. 2016 May 31;6:27022. doi: 10.1038/srep27022 (PMC4886643; doi:10.1038/srep27022)
Supplement: Supplementary Information [file srep27022-s1.doc]

**Encoding****, training and retrieval in ferroelectric tunnel junctions**

Hanni Xu^1^, Yidong Xia^1^†, Bo Xu^1^, Jiang Yin^1^, Guoliang Yuan^2^, and Zhiguo Liu^1^†

^1^National Laboratory of Solid State Microstructures, Collaborative Innovation Center of Advanced Microstructures and Department of Materials Science and Engineering, College of Engineering and Applied Science, Nanjing University, Nanjing 210093, China

^2^School of Materials Science and Engineering, Nanjing University of Science and Technology, Nanjing 210094, China

†To whom correspondence should be addressed. E-mail: xiayd@nju.edu.cn (Y.D.X.); liuzg@nju.edu.cn (Z.G.L.).

**1. The cross-sectional morphology of BTO/LSMO/STO heterostructure.**

**Figure S1** shows dark-field electron microscopy images identifying the heterostructure. The three layers of BTO, LSMO and STO can be discriminated by the different contrast. It clearly reveals that LSMO and BTO layers are fully commensurate with STO substrates. The corresponding FFT pattern demonstrates the crystalline structure of BTO and LSMO layers.


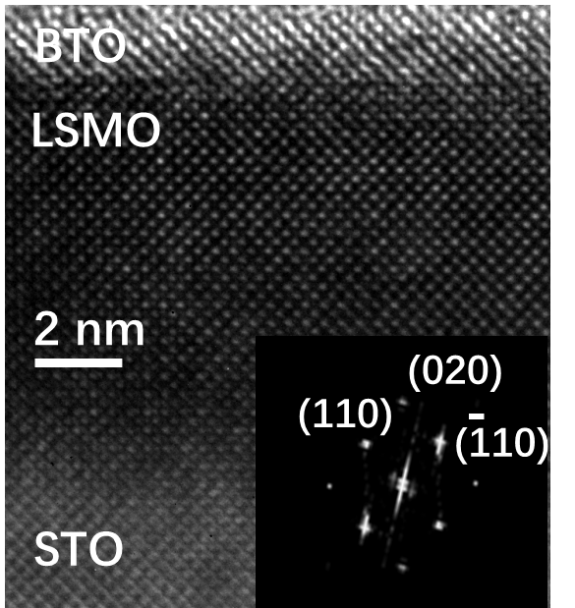


**Figure S1.** The dark-field image of BTO/LSMO/STO heterostructure and the corresponding fast Fourier transformation pattern.

**2. Ferroelectricity of ultrathin BTO and correlation between polarization reversal and resistance switching.**

The local ferroelectric feature of BTO film is confirmed by PFM analysis. **Figure S2a** shows the hysteresis phase loop and the butterfly-like amplitude loop, exhibiting the ferroelectric polarization switching. The phase difference between the two polarization states is about 180^o^. The local coercive voltages are about +1.4 and -1.6 V respectively, as indicated by the minima of the amplitude loop. **Figure S2b** shows the out-of-plane PFM phase image switched by $\pm$3 V. The 180^o^ phase contrast reveals that the polarization is antiparallel in the two domains. Current mapping over the two antiparallel ferroelectric domains is shown in **Fig. S2c**. The region of downward polarization exhibits larger conductivity than that of upward polarization, demonstrating the polarization-dependent TER effect in our BTO FTJs.


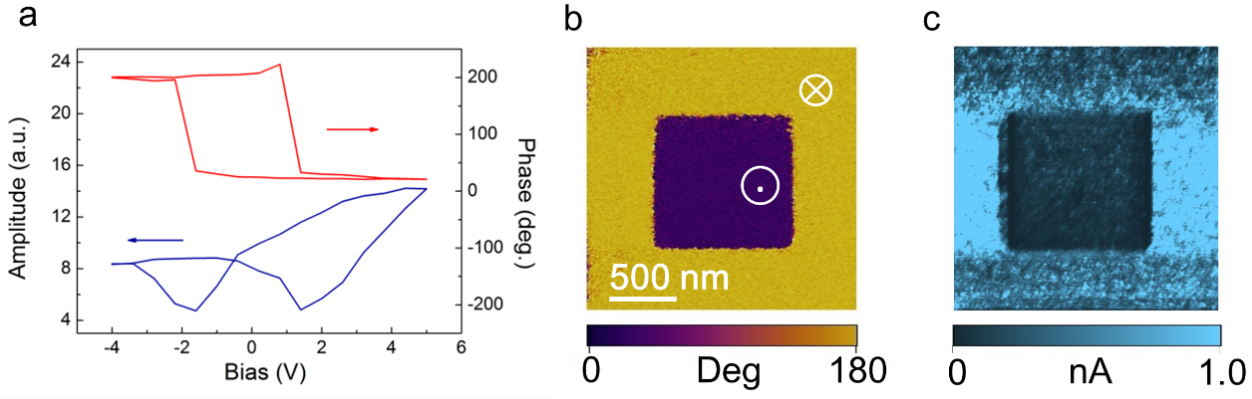


**Figure S2.** The local ferroelectric feature of ultrathin BTO films. **a**, Phase and amplitude loops. **b**, PFM out-of-plane phase image recorded after writing the central area of $1\times1$ $\mu m^{2}$with -3 V and the rest with +3 V. **c**, Current mapping over the same region shown in (**b**), demonstrating the polarization direction-dependent conduction in BTO FTJs.

**3. The detailed decay of “NJU” encoded by using inputs composed of single pulse.**

There are two "00", six "01", three "10" and one "11" used in defining "NJU", as shown in **Fig. 2a**. **Figure S3**, the detailed figure from **Fig. 2b**, summarizes the retention properties of all these 12 codes. The TER values resulted from larger voltages display slight variations (**Fig. S3a**), where TER decreases by about 2% for -4 V and increases by about 10% for +4 V. But, lower voltage pulses ($\pm$2 V) yield drastic changes (**Fig. S3b**), where TER decreases by about 40% for -2 V and increases by about 200% for +2 V. The statistical data from 10 operations are also shown in **Fig. S5**.


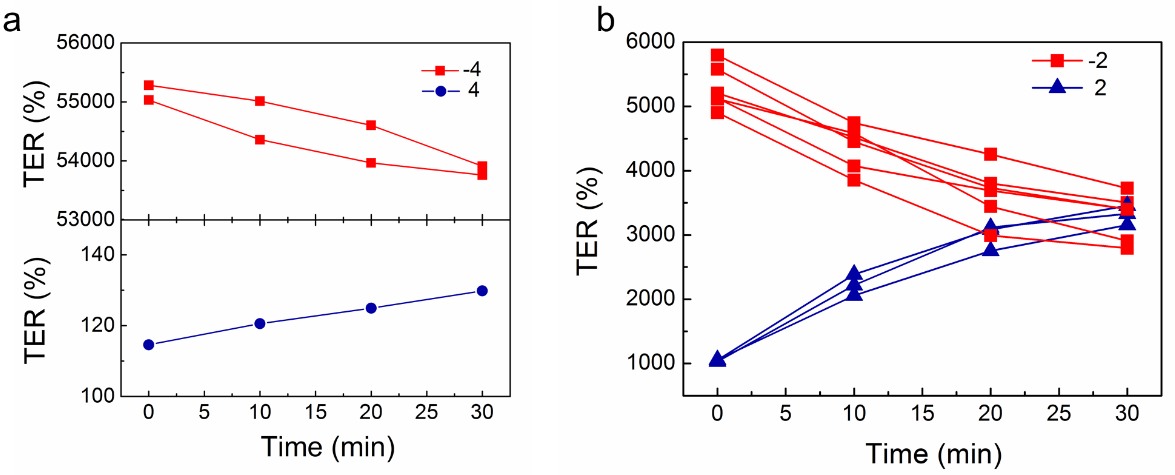


**Figure S3**. The time-dependent TER after encoding by single pulse. **a**, TER from encoding inputs of $\pm$4 V. **b**, TER from encoding inputs of $\pm$2 V.

**4. *n*-dependent relaxation behaviors of samples with Pt top electrodes.**


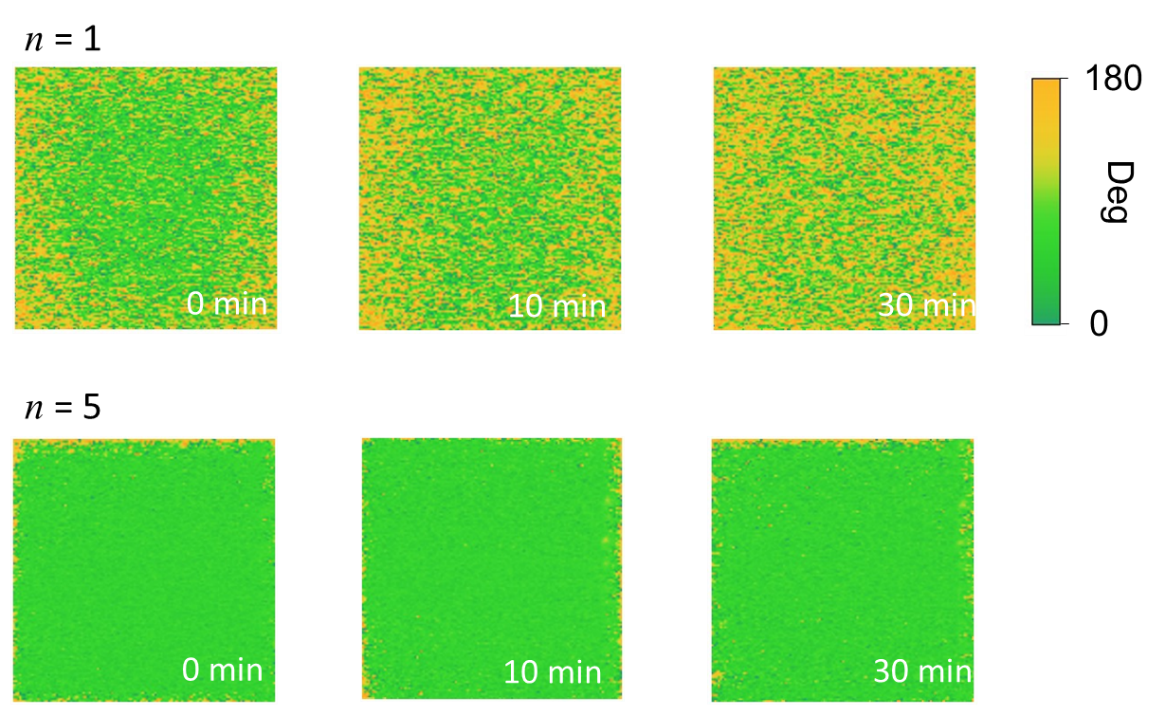


**Figure S4.** PFM mappings with top electrode holding for 0 min, 10 min and 30 min after applied stimuli with different stimuli number.

One stimulus switches most part of the polarization region upward. These upward domains decay a lot with the lapse of time. Almost all the domain reverses into upward after applying 5 stimuli but they can be held without no indication of degradation within the measurement duration. These results conform to the PFM data without top electrode (Figure 4b and Figure S6), indicating that the polarization relaxation under different stimuli number is irrelevant to the top electrode.

**5. Statistical data of variations in TER after 30 min.**

**Figure S5** gives the statistical data of changes in TER from 10 operations, drawing a comparison between encoding "NJU" by single pulse (blue) and training pulse set (red). There are two things of note. First, for encoding by single pulse, TER values from $\pm$2 V exhibit strong time-dependent behaviors than those from $\pm$4 V, in agreement with the results shown in **Fig. S3**. Second, such decay of TER is greatly suppressed when encoding operations are implemented using a training pulse set.

***
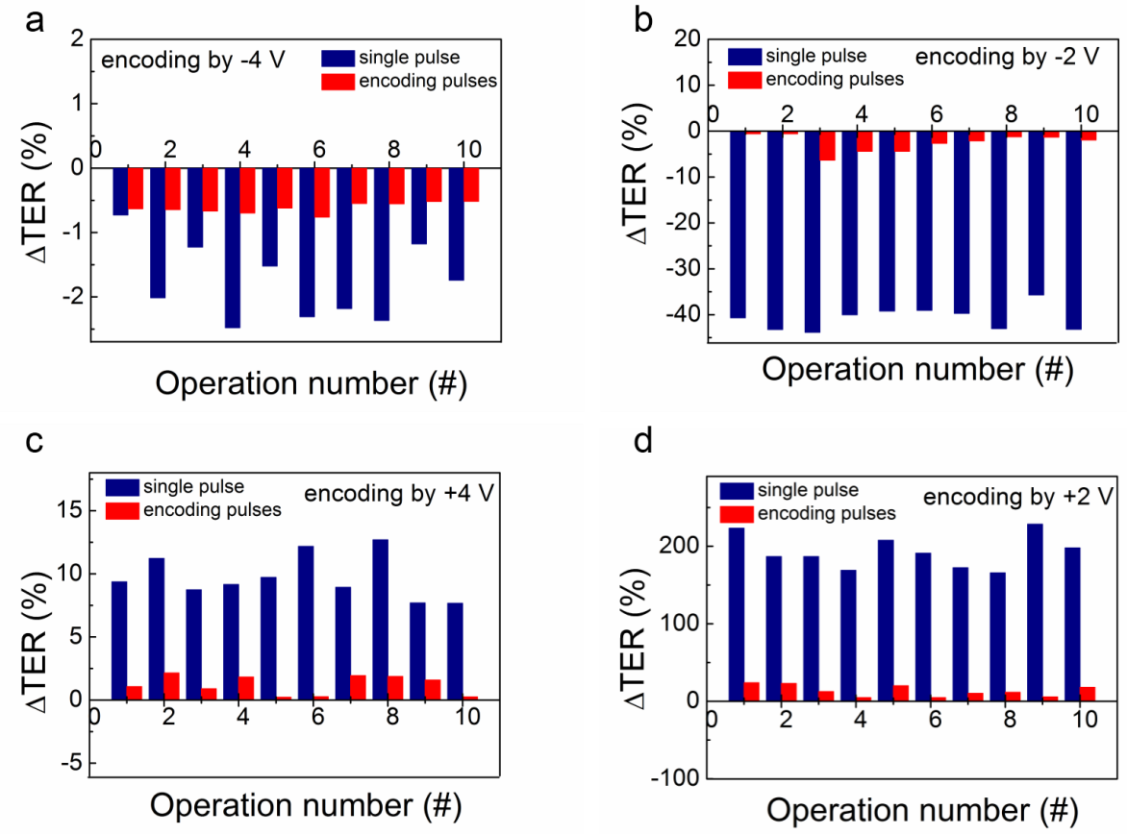
***

**Figure S5**. Statistical data of variations in TER from 10 operations of encoding “00”, “01”, “10” and “11” by inputs composed of single pulse (blue) and training intermittent pulses (red). The pulse parameters are described in **Fig. 1**. $\Delta TER$ is the change of TER after 30 min, i.e. $\Delta TER=[TER\left(30\min\right)-TER\left(0\right)]/TER\left(0\right)$.

**6. Detailed *n*-dependent relaxation behaviors of samples without top electrodes**


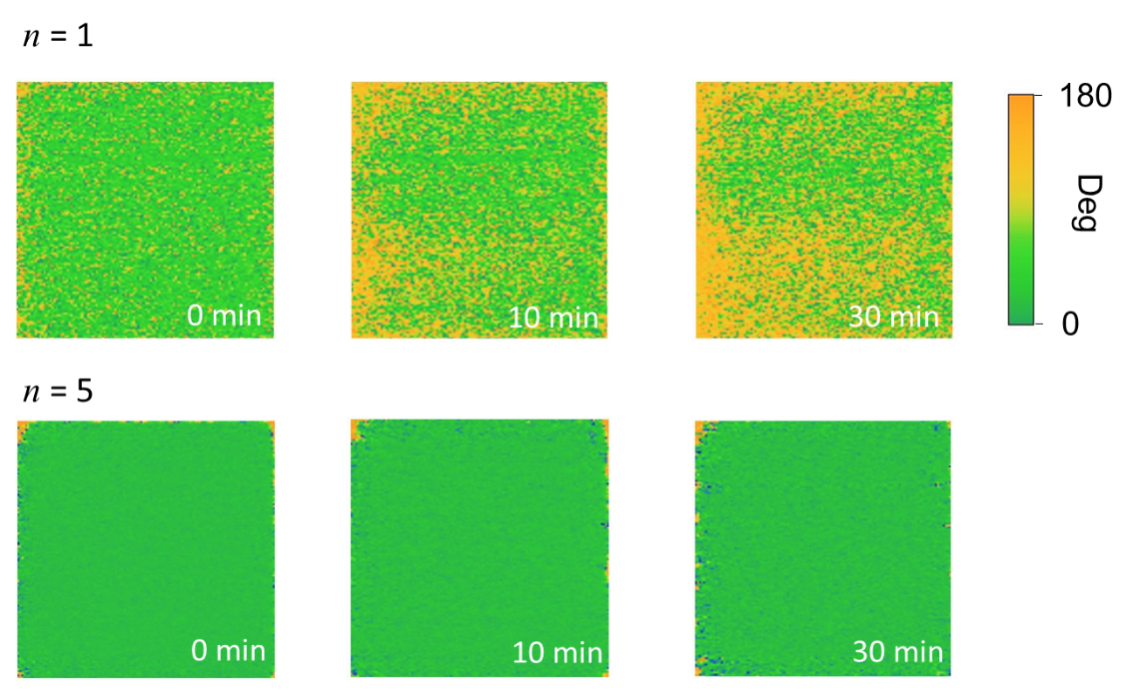


**Figure S6.** PFM images with enhanced resolution after applied stimuli voltage with different stimuli number.

We reduce the scanning frequency and increase the scanning point in PFM experiments to raise the image resolution (higher PFM resolution than that in Figure 4b). Some downward domains still exist in polarization region by one stimulus, indicating that the polarization is not fully switched during the encoding by -2 V. As increasing the stimuli number to 5, the image shows almost uniquely upward polarization.

**7. Methods to program the designed $3\times3$ pattern for PFM experiments.**


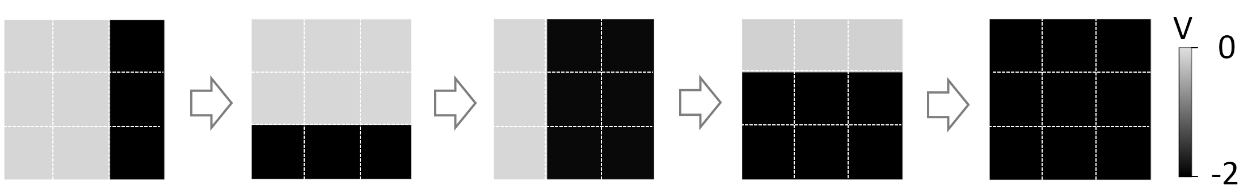


**Figure S7**. Five writing steps are implemented in sequence to achieve the designed $3\times3$ pattern shown in **Fig. 4a**. Black regions are programmed with -2 V and grey regions are written with 0 V.

The $3\times3$ pattern was designed on purpose in order to present a direct perception of different relaxation behaviors in 9 squares electrically written with different history. For example, the number of stimulus changes from 1 (IA) to 5 (IIIC). Different time intervals are also accomplished in the regions with the same number, such as the diagonal “IC🡪IIIA” showing the variation of time intervals. If we did the PFM experiments through the top electrode, we would not get all the relaxation information from different stimuli conditions in one location and at the same time. Because only one stimulation condition can be electrically written through top electrode at a time.

**8. The comparison between training pulse set and one continuous pulse with the equivalent total duration.**

The pulse duration also has impact on the retention properties. However, the training pulse set has its superiority over one continuous pulse even with the equivalent total duration. **Figure S8** compares the barrier profiles resulted from these two types of encoding inputs. After 30 min, the lower $\Phi^{-}$ from one single long pulse reveals that it is less competent than the training pulses.

***
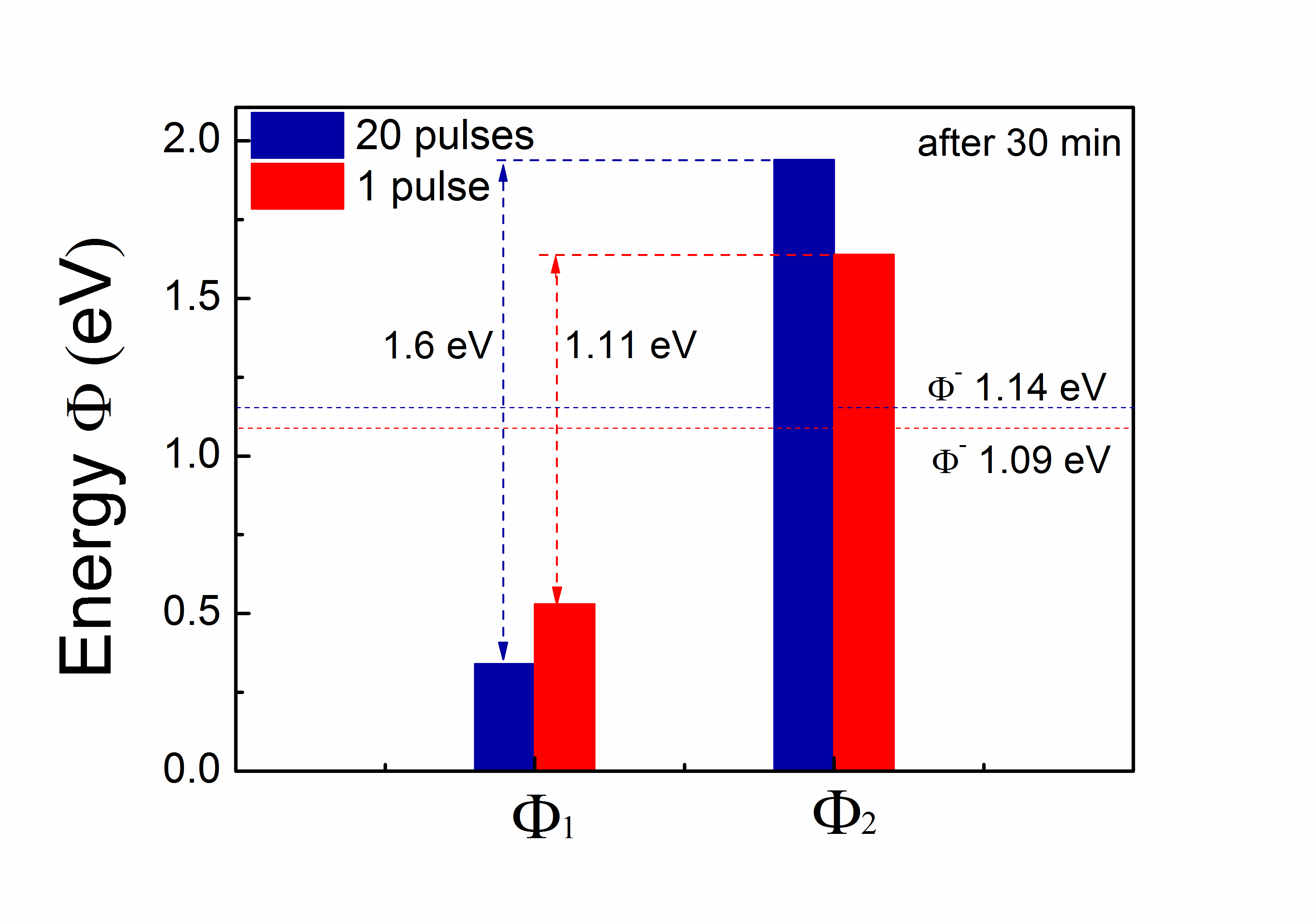
***

**Figure S8**. The potential energies from two kinds of encoding inputs after 30 min. Blue: training input set composed of 20 pulses. Each pulse is -2 V in amplitude, 200 $\mu s$ in duration, and 1 ms in interval. Red: input of one single pulse with -2 V and 4 ms width that is equivalent to the total integrated excitation time of the pulse set.
